# Resveratrol and 1,25-dihydroxyvitamin D decrease Lingo-1 levels, and improve behavior in harmaline-induced Essential tremor, suggesting potential therapeutic benefits

**DOI:** 10.1038/s41598-024-60518-4

**Published:** 2024-04-29

**Authors:** Zeynab Pirmoradi, Mohsen Nakhaie, Hoda Ranjbar, Davood Kalantar-Neyestanaki, Kristi A. Kohlmeier, Majid Asadi-Shekaari, Amin Hassanshahi, Mohammad Shabani

**Affiliations:** 1 Neuroscience Research Center, Institute of Neuropharmacology, Kerman University of Medical Sciences, Kerman, 76198-13159 Iran; 2 Gastroenterology and Hepatology Research Center, Institute of Basic and Clinical Physiology Sciences, Kerman University of Medical Sciences, Kerman, Iran; 3 Medical Mycology and Bacteriology Research Center, Kerman University of Medical Sciences, Kerman, Iran; 4 Department of Drug Design and Pharmacology, Faculty of Health Sciences, University of Copenhagen, Copenhagen, Denmark; 5 Department of Physiology, Medical School, Bam University of Medical Sciences, Bam, Iran

**Keywords:** Essential tremor, Resveratrol, 1,25-Dihydroxyvitamin D, Harmaline, Sirt1, Neuroscience, Physiology, Diseases, Medical research, Neurology, Signs and symptoms

## Abstract

Essential tremor (ET) is a neurological disease that impairs motor and cognitive functioning. A variant of the Lingo-1 genetic locus is associated with a heightened ET risk, and increased expression of cerebellar Lingo-1. Lingo-1 has been associated with neurodegenerative processes; however, neuroprotection from ET-associated degeneration can be conferred by the protein Sirt1. Sirt1 activity can be promoted by Resveratrol (Res) and 1,25-dihydroxyvitamin D3 (VitD3), and thus these factors may exert neuroprotective properties through a Sirt1 mechanism. As Res and VitD3 are linked to Sirt1, enhancing Sirt1 could counteract the negative effects of increased Lingo-1. Therefore, we hypothesized that a combination of Res-VitD3 in a harmaline injection model of ET would modulate Sirt1 and Lingo-1 levels. As expected, harmaline exposure (10 mg/kg/every other day; i.p.) impaired motor coordination, enhanced tremors, rearing, and cognitive dysfunction. When Res (5 mg/kg/day; i.p.) and VitD3 (0.1 mg/kg/day; i.p.) were given to adult rats (n = 8 per group) an hour before harmaline, tremor severity, rearing, and memory impairment were reduced. Individual treatment with Res and VitD3 decreased Lingo-1 gene expression levels in qPCR assays. Co-treatment with Res and VitD3 increased and decreased Sirt1 and Lingo-1 gene expression levels, respectively, and in some cases, beneficial effects on behavior were noted, which were not seen when Res or VitD3 were individually applied. Taken together, our study found that Res and VitD3 improved locomotor and cognitive deficits, modulated Sirt1 and Lingo-1. Therefore, we would recommend co-treatment of VitD3 and Res to leverage complementary effects for the management of ET symptoms.

## Introduction

Essential tremor (ET) appears as a moderately asymmetric tremor with a frequency of 4–12 Hz during voluntary movements^1^ and is characterized by bilateral postural and kinetic tremors in the upper limbs, however uncontrolled movement can also occur in other areas of the body^2,3^. ET is one of the most common neurological disorders as it occurs almost 8 times more frequently than Parkinson's disease and exerts enormous economic and social effects^4,5^. Roughly 0.9% of people worldwide have ET, with more than 4% of them exhibiting tremor beyond the age of 65.

The origin of ET disease remains unknown^5^. ET has been linked to various mechanisms, including expression of the Lingo-1 gene^6^. Sequence variants of the gene for Lingo-1 have been identified as risk factors for ET^7^. Lingo-1 is a protein that contains leucine-rich repeat domains as well as an immunoglobulin G domain^8,9^. The activation of this protein in neurons causes the development cone to collapse resulting in inhibition of growth and repair of neural pathways and axons^10,11^. Increases of expression of Lingo-1 occur in the cerebellar cortex of ET patients, leading to oligodendrocyte immaturity and Purkinje cell degeneration, and targeting its expression has been suggested as a treatment for ET^6,11,12^. While inhibiting Lingo-1 could be useful in the management of ET, few studies have evaluated how to control the expression of this gene while monitoring ET outcomes.

Another treatment approach for ET could be the use of antioxidants, which have been shown to be neuroprotective in a process involving activation of sirtuins. The sirtuin family plays an important role in apoptosis, metabolism, neurite outgrowth, axon development, and proliferation^13,14^. Sirtuin-1 (Sirt1) is the earliest recognised member of the sirtuin family and is a major protein deacetylase that controls a wide range of physiological processes, notably neuroprotection, oxidative stress, inflammation, and mitochondrial functioning^15^. Sirt1 is highly expressed in the white matter of the CNS, hippocampus, cerebellum, and hypothalamus^14^. Several studies have shown that Resveratrol (Res) activates Sirt1, and consistent with this activation, Res contains anti-inflammatory and pain-relieving properties and can protect nerve fibers from oxidative stress, toxicity, and cell degeneration^16,17^. Sirt1 is also controlled by another target suggested as relevant for the treatment of neurodegeneration, 1,25-dihydroxyvitamin D (VitD3). VitD3 is a fat-soluble vitamin that when produced, functions as a hormone^18^ and regulates neurotrophins, neuronal differentiation, and neuronal maturation^19^. Calcitriol, also known as D (OH2)1,25, is the active form of VitD3^18^. Neurons and glial cells express VitD3 receptors with the highest levels seen in the cerebellum, hippocampus, hypothalamus, and substantia nigra^20,21^. VitD3 controls the concentration and activity of human endothelium Sirt1 via increasing hydrogen peroxide^22^. In addition, VitD3’s anticancer effects result from the interplay of the vitamin D receptor (VDR), activation of phosphorylation, and stimulation of Sirt1^14,22^, and VDR deacetylation is mediated by Sirt1 and enhanced by Res through activation of Sirt1^23^. Hence, Res and VitD3 share fundamental biochemical, biological, and signaling pathways resulting in anti-inflammatory and antioxidant effects, and these features may play a role in improving ET degeneration^24^.

As enhancing Sirt1 could counteract the negative effects of increased levels of Lingo-1, which has been shown to be aberrant in ET, and effects of Res and VitD3 involve Sirt1, we wished to examine whether a pharmacological combination of Res-VitD3 could alter levels of Sirt1 and Lingo-1, induce neuroprotection, and improve ET-associated behaviors. One commonly used animal model for the study of potential neuroprotective agents for ET is the harmaline-injected rat. Harmaline is a tremorgenic alkaloid that has been extensively studied in animal models of ET because its effects are similar to those of centrally generated tremors in rodents^25^. Experimental animals are given harmaline, which causes them to tremor uncontrollably by boosting glutamate discharge in climbing fibers, leading to severe damage to the Purkinje cells and other parts of the olivocerebellar pathway^26^. This model is well suited to evaluate potential neuroprotective strategies for ET. Consequently, we employed the harmaline model of ET to explore the behavioral and cellular effects of a pharmaceutical combination of Res-VitD3. Moreover, we sought to determine whether alterations in Sirt1 and Lingo-1 expression are correlated with noted behavioral and cellular changes.

## Material and methods

### Animals and experimental design

Male Wistar rats aged eight weeks (200–250 g) were used in this study. Animals were kept in individual cages with access to food and water ad libitum and maintained in a room with sound insulation, a recorded temperature of 24 ± 1 °C, and a closely monitored relative humidity of 55 ± 5% on a 12-h dark/light cycle. Every effort was made to minimize animal suffering during all stages of the study. All procedures were approved by the Kerman Medical University Ethics Committee (IR.KMU.AH.REC.1400.194). The animals were distributed into 9 groups (n = 8), including controls. The control groups were: Saline (S), (Saline + Res (SR)), (Saline + VitD3 (SV)), (Saline + Res + VitD3 (SRV)), and (10% ethanol (E)). The ET model group was (harmaline + Saline) (HS), and the ET model treatment groups were (HR), (HV), and (HRV). In the ET model groups, Res (5 mg/kg, i.p.)^27^ or VitD3 (0.1μg/kg, i.p.)^28^ were administered 60-min prior to harmaline (10 mg/kg, i.p.) once a day for 3 separated days (day 1, day 3, and day 5). The animals were transported to the examination room for adaptation one hour prior to the conduction of behavioral tests. In the HRV group, the rats received co-treatment of Res and VitD3 60-min before harmaline once a day for 3 separate days. Each group was given a 15-min break in between each of the four behavioral tasks (tremor score evaluation, open field test, wire grip, rotarod, and gait analysis). The learning part of the passive avoidance task was conducted 3 h after the tremor symptoms had ceased. Memory retrieval was assessed in the passive avoidance task 24 h later. Following 24 h after the last experiments, the animals were deeply anesthetized by isoflurane, the heart was perfused with saline and Buen fixative, and the brains were removed. The cerebellum was rapidly separated, and brain samples (n = 4) of each group were fixed in 10% formalin and 2.5% glutaraldehyde solution for histological tests^29^. For molecular tests, 4 animals were euthanized by cervical dislocation, and the cerebellum was rapidly separated and kept at − 80 °C.

### Drugs

In this study, 10 mg/kg of harmaline hydrochloride dihydrate (Sigma, cat number: H1392) was given intraperitoneally (i.p.) on the first, third, and fifth days to elicit Essential tremor. In previous studies, a single dose of 4–30 mg/kg of harmaline produced tremors in rats^26,30^; whereas, expression of Lingo-1 was found to be altered following 5 days of treatment^31^. As we wished to evaluate levels of Lingo-1, harmaline was administered in 3 doses over a period of 3 days, with an incrementing interval of 10 mg/kg resulting in a final dose of 30 mg/kg^32,33^.

**Figure 1:**
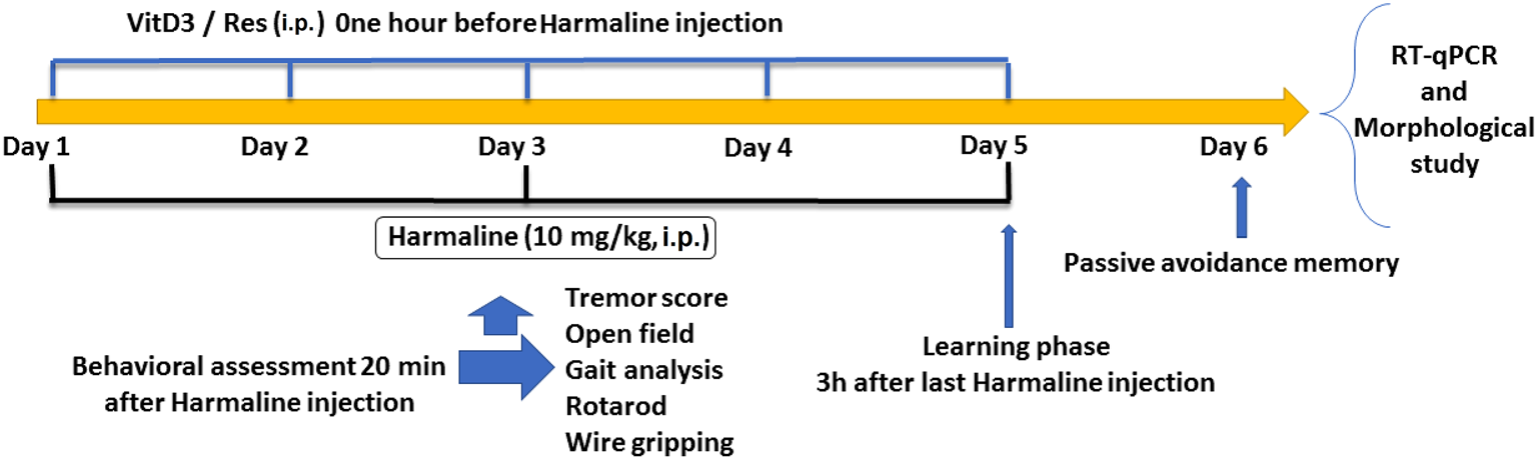
A timeline detailing the time points for behavioral/molecular/histological tests.

Res was purchased from Solarbo Co. in China (cat number: R8350) and dissolved in 10% ethanol. For 5 days, a dosage of 5 mg/kg/day of Res was administered (i.p.) into the target groups. VitD3 was purchased from healthaid.co.uk (health certificate: 5019781010424) and given i.p. at 0.1 µg/kg/day (dissolved in 10% ethanol) for 5 days^34^. The doses of Res and VitD3 were selected based on research studies conducted in the field of neurodegenerative diseases, including Parkinson's disease. Furthermore, a pilot study was conducted as a means of validating the dosages utilized in previous studies for the purposes of this particular investigation^34–36^. The timeline of drug administration, behavioral testing, and histological analysis for our study is depicted in Fig. 1.

### Behavioral tasks

#### Tremor scoring

The occurrence of tremors was evaluated by two observers who were blinded to treatment. The following quantitative tremor scoring was used to analyze the data collected in the open field test 20-min after harmaline injection. The severity of tremor was assessed using a categorical scale that scored the degree of tremor based on observable characteristics as follows: 0: Without any tremor, 1: Mid: Tremor just in head and neck (occasional), 2: Intermittent: Tremor present in all body parts (occasional), 3: Persistent: Tremor present in all body parts, including the tail (persistent), 4: Severe: Inability to stand and/or walk due to tremor (persistent). A tremor classified as intermittent (2) refers to tremor episodes that occur occasionally in all body parts. On the other hand, a tremor classified as persistent (3) means that the tremor is consistently present across all body parts and may continue for a longer duration^32^*.*

#### Open field test

The open field arena was made of Plexiglas, measuring 90 cm length × 90 cm width × 45 cm height. There were a total of 16 squares spread throughout the building's floor, with 8 each in the building's center and outside. Each rat was put in the center of the arena at the start of the session, and its behavior was recorded and analyzed offline during a 5-min period (Ethovision 7.1, Noldus Information Technology, Netherland). The number of groomings (a sign of anxiety-like behavior), rearings (as a measure of vertical activity and exploratory behaviors), duration of relative time spent in central and peripheral zones (CZD and PZD) (as a sign of anxiety-like behavior), as well as the total distance moved (TDM) were measured. To disinfect the arena after each experiment, 70% ethylic alcohol was used^37,38^.

#### Footprint

The footprint test was performed to analyze the gait kinematics and walking style of the rats. The rats' hind limbs were dyed with non-toxic ink, and they were given access to a 100 cm length × 10 cm width × 10 cm height Plexiglas tunnel that led to a darkened cage. The bottom of the Plexiglas tube was lined with a sheet of white absorbent paper (100 cm × 10 cm). The analysis of footprint patterns focused on three step parameters (cm): (a) Right stride length, (b) Left stride length, and (c) stride width. To evaluate these parameters, we selected an average sequence of three consecutive steps for each animal. The stride length was calculated by measuring the distance between the centers of each paw print on one side of the body at a right angle to the other, and the hind paw stride width was calculated by measuring the same distance on the other side of the body. Both the first and last footprints of each trail were disregarded^37^.

#### Wire grip test

The muscular strength and equilibrium of the rats were evaluated with a wire grip test. For this, rats were suspended between two platforms using a horizontal steel wire (80 cm long, 7 mm in diameter). The wire employed for the suspension of the animals was situated at a height of roughly 50 cm above a padded base. This elevation was selected with the intention of impeding the animals from leaping downwards, while also guaranteeing their safety in the event of a descent. The rodent stood upright with its front feet together as it clung to the wire, and three sessions were conducted on each rat with a 5-min interval. The latency to fall for each session was timed using a stopwatch^37,39^.

#### Rotarod performance test

An accelerated rotarod was used to assess each animal's motor coordination and balance. All animals were given a day of pre-test training. The animal was first placed on a rod and rotated at speeds ranging from 10 to 60 revolutions per min. A time limit of 300 s and a 5-min break between 3 trials were used to determine the average animal stay time^40^.

#### Passive avoidance test

The rat’s associative memory and learning were examined with a passive avoidance test using a shuttle box. An animal that has experienced a negative response to a specific stimulus in the past will learn to avoid that stimulus and its surrounding environment. The shuttle box was divided into light and dark compartments by a door and measured 100 cm length × 25 cm width × 25 cm height. The rods in the chamber's floor were connected to a shock generator. The rats were habituated to the device by being exposed to it for an hour before the actual test. Each rat was given 30 s to investigate the dark room after being placed in the bright area. After waiting 1 h, the rat was shocked electrically (0.5 mA, 2 s) when it entered the dark room from the light room. The process was repeated up to five times at 1 h intervals until the animal learned to avoid the dark chamber. The testing and grading phases occurred 24 h following the study and preparation phases. Step-through latency (STL) was determined by placing the animal in the light chamber with the door closed, waiting 10 s, and then opening the door. Time spent in the dark chamber (TDC) was also measured for a period of 5 min following the door opening^41^.

### Detection of Lingo-1 and Sirt1 Genome by Reverse Transcription-Quantitative Polymerase Chain Reaction (RT-qPCR)

Cerebellum tissues were rapidly extracted after animal sacrifice. The tissues were quickly frozen in liquid nitrogen and kept at − 80 °C. Total RNA was isolated from cerebellum tissues using the RNA Kit (SinaPure-RNA-EX6031) as per manufacturer instructions. The concentration and purity of the obtained RNAs were measured by a BioPhotometer (NanoDrop™, N-D 2000, Thermo Scientific, USA). RNA integrity was evaluated by electrophoresis of the RNA on an agarose gel (18 srRNA and 28 srRNA bands). In order to investigate the presence of the Lingo-1 and Sirt1 genomes, the extracted RNAs were subjected to cDNA synthesis using the AddScript cDNA Synthesis Kit (Add Bio, Korea). Briefly, in this reaction, 1 to 5 μg (based on concentration) of each sample along with 1 μL of 50 μM random hexamer, 13.4 μL of diethyl pyrocarbonate (DEPC) water, 1 μL of deoxynucleotide triphosphate (dNTPs) (10 mM), 0.5 μL of the RNase inhibitor (20 units), 1 μL and reverse transcriptase (200 units) were prepared in a total volume of 20 μL and incubated according to the temperature cycle. Synthesized cDNAs were stored at − 70 °C until further analysis by a real-time method. The mixture of each reaction included 12.5 µL of RealQ Plus 2 × Master Mix, 0.5 ml of each primer, 10.5 µL of PCR-grade H2O, and 1 µL of cDNA in a final volume of 25 µL. The PCR reaction was performed by using the following thermal cycling conditions: 15-min at 95 °C followed by 40 cycles at 95 °C for 15 s, 59  °C for 30 s, and incubation at 72 °C for 30 s, for a total run time of about 2 h. Primers to amplify Lingo homologs were mLingo-1For, 5′-TCTATCACGCACTGCAACCTGAC-3′, and mLingo-1Rev, 5′- AGCATGGAGCCCTCGATTGTA-3′. Primers for Sirt1 were mSIRT-1For, 5′-TGCTGGCTTAATAGACTTGCA-3′, and mSirt1Rev, 5′- CACCGTGGAATATGTAACGA-3′, GAPDH For, 5′-ATGGAGAAGGCTGGGGCTCACCT-3′, and GAPDH Rev 5′-AGCCCTTCCACGATGCCAAAGTTGT-3′. Glyceraldehyde 3-phosphate dehydrogenase gene was used as an internal control. The relative changes in target genes expression versus a reference gene were determined according to the 2^-ΔΔCT^ method^42^.

### Morphological analysis

#### Hematoxylin–Eosin staining

To analyze the morphology of Purkinje cells, we conducted a histological examination of cerebellar tissue to assess cell survival and observe the characteristics of the nucleus and cytoplasm. The Hematoxylin–Eosin (H&E) staining method was utilized for this purpose.

Coronal sections with a thickness of 5 μm, were obtained from the cerebellum cortex of each animal at 25 μm intervals. These sections were prepared using a semi-automated microtome (Leica RM2145) and then transferred onto gelatinized slides. To prepare the sections for analysis, dewaxing by immersing the slides in xylene for 5 min, followed by rehydration in a graded ethanol series (100%, 95%, 70%, 50% ethanol, 5 min each) was conducted. H&E staining was performed according to standard protocols (Fischer et al., 2008). Briefly, slides were stained in Harris hematoxylin solution for 5 min to stain nuclei blue/black, differentiated in 1% acid alcohol, and counterstained in eosin-phloxine solution for 30 s to stain cytoplasm and extracellular matrix pink^43,44^. For image acquisition, a Nikon 50i plus microscope with magnification of 400×, along with a Nikon Axiocam camera was employed. Only Purkinje cells with distinct nuclei and nucleoli were considered as healthy, living cells for evaluation. To quantify the number of Purkinje cells in each area, we analyzed at least four randomly assigned fields per animal. In each experimental group, the calculation of Purkinje cell count was performed based on these measurements (n = 4 in each group)^45^.

#### Nissl staining

Purkinje cell degeneration in the cerebellar cortex was analyzed by standard methods^45^. Briefly, paraffinized tissue containing the cerebellum was cut into 5 µm sections using a semi-automated microtome (Leica RM2145), and the sections were stained with cresyl violet (Nissl staining)^46^. For every animal (n = 4 per group), the percentage of Purkinje cell degeneration in four different regions of each cerebellar slide's coronal section was calculated. The slides of cerebellum for each animal were observed by two researchers under a Nikon 50i plus microscope, and a Nikon Axiocam camera was used for image acquisition. According to the samples stained with cresyl violet, nomal Purkinje cells exhibit distinct organelles and nucleus, clear cytoplasm, and prominent nucleolus. In contrast, the degenerated cells have shrinkaged nuclei and dark cytoplasm.

In both H&E staining and Nissl staining, the percentage of degeneration is typically calculated by counting the number of degenerated cells and comparing that number to the total number of cells in the sample. To compare the percentage of degeneration between the groups, the total number of degenerated cells and the total number of cells in the sample (degenerated and healthy) were counted. Percentage of degenerations = ((Number of degenerated cells/Total number of cells) * 100)^45^.

#### Ultrastructural study

For the ultrastructural study, specimens (cerebellar cortex) were fixed in 2.5% phosphate-buffered glutaraldehyde (pH 7.4) at 4 °C and then post-fixed in 1% osmium tetroxide in the same buffer, dehydrated, and embedded in resin. Ultrathin sections (70 nm) were stained with uranyl acetate and lead citrate and photographed using a transmission electron microscope (TEM, Zeiss EM 10) at the TEM department of the Kerman Neuroscience Research Center^47^.

### Statistical analysis

GraphPad Prism 8 (GraphPad Software, USA) was used for statistical analysis of data and figure production. All data were first assessed for normality using the Kolmogorov–Smirnov test. Results found to be normally distributed (*p* > 0.05) were expressed as mean ± SD and analyzed using a one-way ANOVA test in control groups. To compare HS with S, unpaired t tests were performed. A two-way ANOVA was conducted between the treated groups and the day of treatment, followed by the post-hoc Tukey's test for comparisons among groups. One-way ANOVA followed by the Game Howell test was used to estimate the interaction between gene and treatment effects in the RT-PCR analysis. Results that failed normality testing (*p* < 0.05 in K-S test) were expressed as median and interquartile range (Scatter dot plots or Box and Whiskers expressed as median (interquartile range)), and analysed using either a Mann Whitney test or a Kruskal–Wallis test. The minimum level of significance was *p* < 0.05.

### Ethical statement

All experiments were done in accordance with the ARRIVE guidelines and National Institutes of Health Guide for the Care and Use of Laboratory Animals (NIH Publication No. 80-23, revised 1996). All procedures were approved by the Research and Ethics Committee of Kerman Universities of Medical Sciences, Kerman, Iran.

## Results

### The tremor symptoms were improved by Res and VitD3

**Figure 2:**
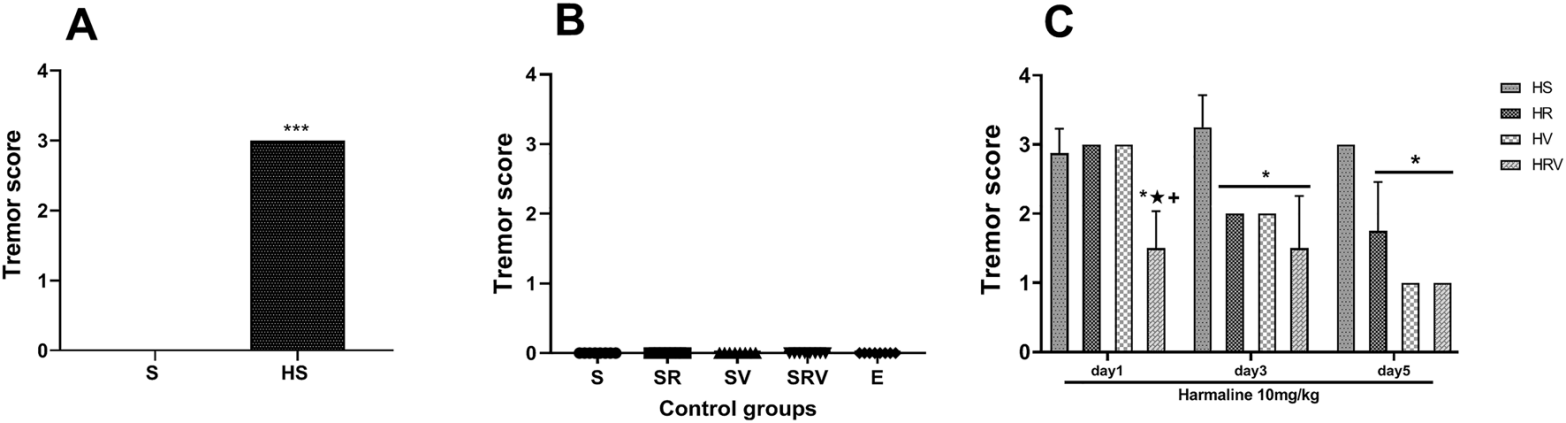
Harmaline significantly increased tremor scale scores in the HS group when compared to scores in the Saline group. The HRV, HV, and HR groups showed lower tremor scores in comparison with scores seen in the HS group on days three and five. Data are expressed as the mean ± SD (* in comparison to the harmaline group, + in comparison to the HV group, ★ in comparison to the HR group). Saline (S), Saline + Res (SR), Saline + VitD3 (SV), Saline + Res + VitD3 (SRV), Ethanol (E), harmaline + Saline (HS), harmaline + Res (HR), harmaline + VitD3 (HV), and harmaline + Res + VitD3 (HRV).

Consistent with a motor effect of harmaline treatment, significantly higher tremor scale scores were exhibited in the HS group when compared to scores in the Saline group (F (6, 56) = 0.04, *p* < 0.001, Fig. 2). Indicating an effect of the co-treatment with the neuroprotective agents, significantly lower tremor scores were seen in the Res + VitD3 group compared to scores in the HS, Res, and VitD3 groups on day one (*p* < 0.05). The Res + VitD3, VitD3, and Res groups also showed lower tremor scores in comparison with scores seen in the HS group on days two and three (Fig. 2).

### Res + VitD3 alleviated anxiety-like behaviors

**Figure 3:**
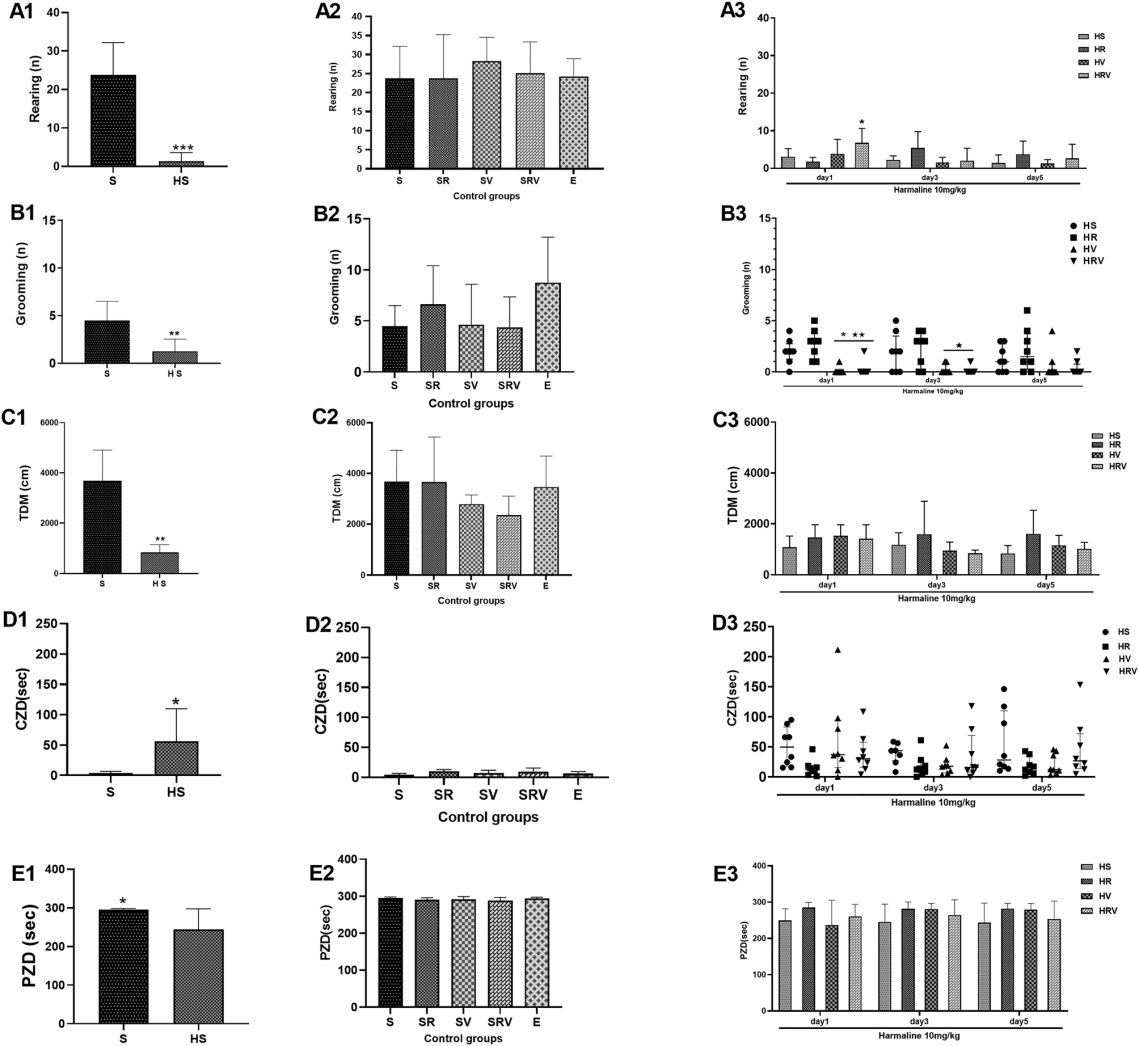
Characterization of the animal’s exploratory activity and anxiety-like behavior in the open field test. (**A**) Frequency of rearing, (**B**) number of grooming sessions, (**C**) total distance walked, (**D**) central zone duration, and (**E**) peripheral zone duration are shown. On day one, rearing was significantly higher in the HRV compared to the HR group. Additionally, on day one, the HRV and HV groups showed significantly lower grooming in comparison to the HS and HR groups. HRV and HV groups showed a decrease in grooming compared to the HR group on days one and three. The HS group showed a lower TDM in comparison to the Saline group. Data are expressed as the mean ± SD (* in comparison to the harmaline group, ★ in comparison to the HR group). Saline (S), Saline + Res (SR), Saline + VitD3 (SV), Saline + Res + VitD3 (SRV), Ethanol (E), harmaline + Saline (HS), harmaline + Res (HR), harmaline + VitD3 (HV), and harmaline + Res + VitD3 (HRV). Data were not normally distributed are represented as medians with interquartile ranges as a scatter dot plot.

On day one, the numbers of rearing (F (6, 56) = 0.003, *p* < 0.05, Fig. 3) were significantly higher in the harmaline-injected animals treated with Res and VitD3 as compared to the HR group. Additionally, HRV and HV groups showed significantly lower grooming in comparison to HS (*p* < 0.05) and HR (*p* < 0.01) groups on day one (F (6, 56) = 0.82, Fig. 3). On days one and three, the HRV and HV groups showed a decrease in the number of grooming sessions when compared to the HR group (*p* < 0.05; Fig. 3). The harmaline-treated group showed a lower TDM when compared to the Saline group (F (6, 56) = 0.40, *p* < 0.01, Fig. 3). However, there was no significant difference between harmaline and the other treatment groups in this parameter (*p* > 0.05, Fig. 3). The animals that received injections of harmaline spent a greater amount of time in the central zone as compared to the group that received Saline injections (Fig. 3). Correspondingly, the harmaline group demonstrated a reduced amount of time spent in the peripheral zone as compared to the Saline group (Fig. 3). Although these findings may suggest a lowered level of anxiety-like behavior in the harmaline group, one confound is that animals experiencing tremors were unable to move or explore the area. The treatment groups showed no significant alteration in comparison to the harmaline group (Fig. 3).

### Res + VitD3 had no effect on gait disturbances

**Figure 4:**
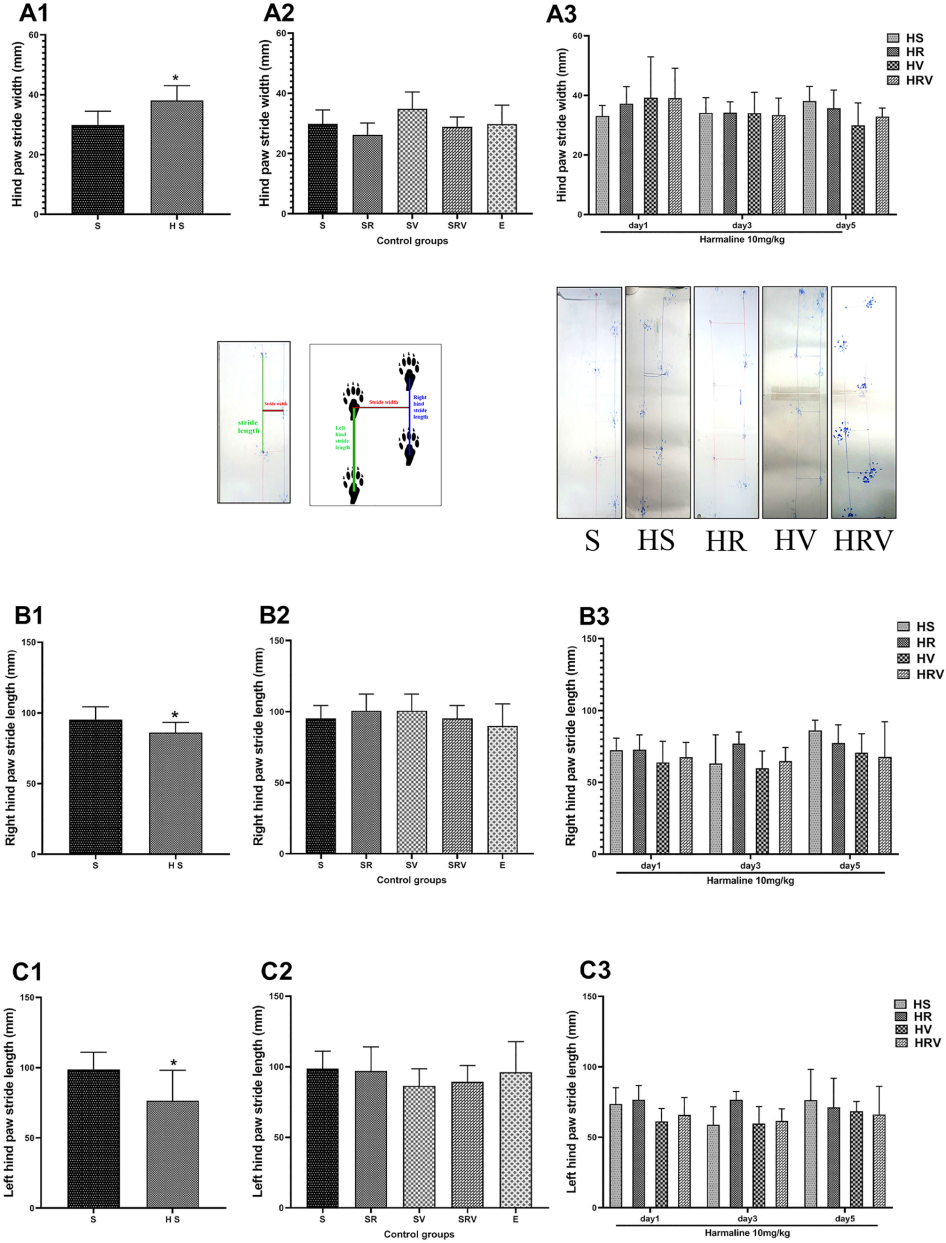
Gait analysis in the footprint test. Results from tests evaluting (**A**) stride width (cm), (**B**) right hind stride length (cm), and (**C**) left hind stride length (cm) are presented. Recorded representative traces of stride length and stride width from animals in Saline, harmaline, and treated groups are shown in the middle panel. The step width was significantly higher in the HS group compared to the Saline group. Left and right step length was significantly smaller in the HS group compared to the Saline group. Data are expressed as the mean ± SD. (* in comparison to the harmaline group). Saline (S), Saline + Res (SR), Saline + VitD3 (SV), Saline + Res + VitD3 (SRV), Ethanol (E), harmaline + Saline (HS), harmaline + Res (HR), harmaline + VitD3 (HV), and harmaline + Res + VitD3 (HRV).

Treatment with harmaline also disturbed gait as the step width was significantly higher in the HS group when compared to that of the Saline group (F (6, 56) = 0.1, *p* < 0.05, Fig. 4). Moreover, harmaline had an adverse effect on left and right step length as demonstrated by significantly decreased left (*p* < 0.05) and right (*p* < 0.05) step length in the HS group when compared to that in the Saline group (Fig. 4). However, no significant differences were found between treatment groups and the HS group, suggesting that Res and VitD3 did not have an effect on gait (Fig. 4).

### Res improved motor coordination and balance

**Figure 5:**
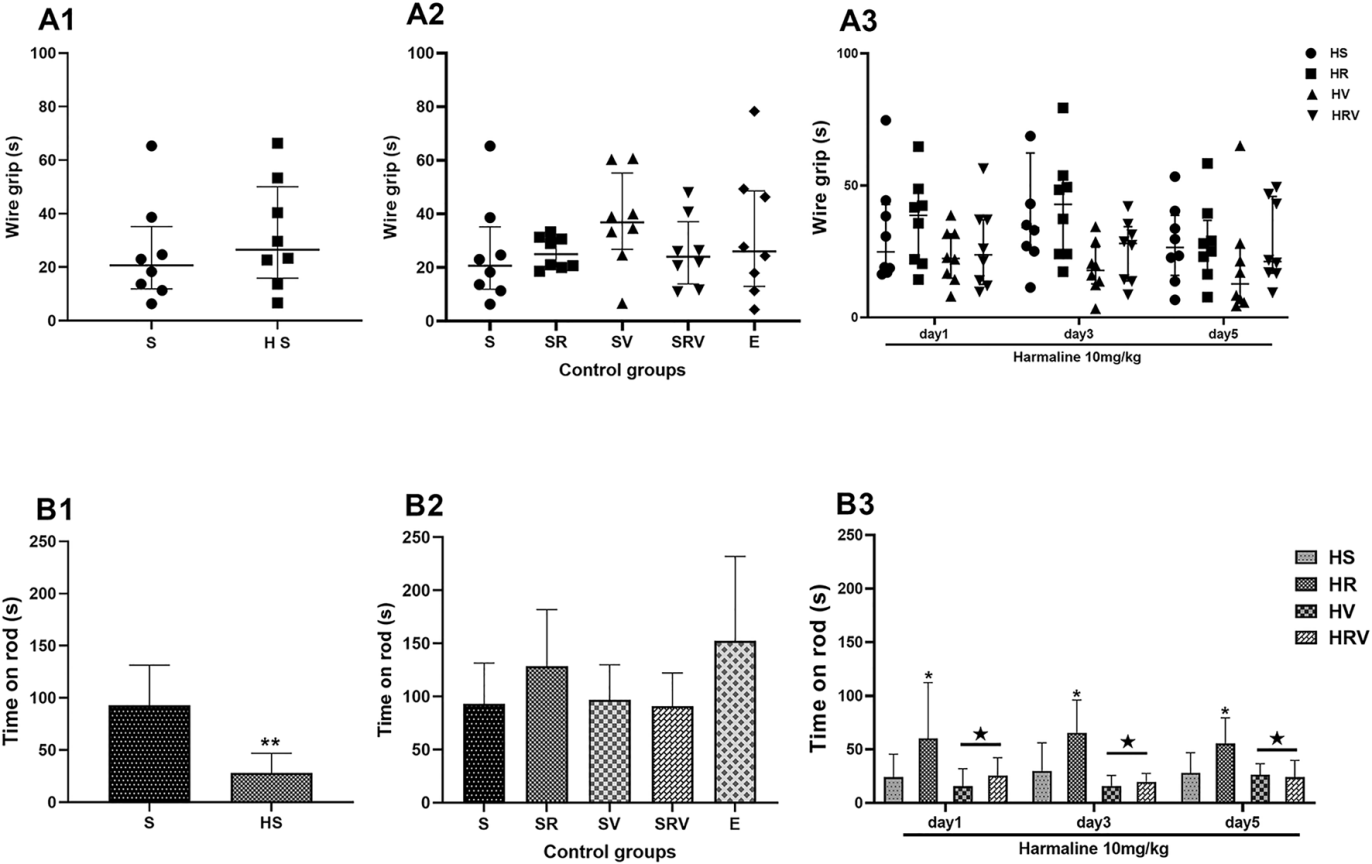
Grip strength and rotarod analysis. Results from A) Wire grip, and B) rotarod tests are shown. In the wire grip test, there was no significant difference between the HS and treatment groups compared to the Saline group. Data are represented as medians with interquartile ranges as a scatter dot plot. The HS spent significantly less time on the rod than the Saline group. HR rats spent a longer time on the rod when compared to the HS group. Data are expressed as the mean ± SD (* in comparison to the harmaline group, ★ in comparison to the HR group). Saline (S), Saline + Res (SR), Saline + VitD3 (SV), Saline + Res + VitD3 (SRV), Ethanol (E), harmaline + Saline (HS), harmaline + Res (HR), harmaline + VitD3 (HV), and harmaline + Res + VitD3 (HRV).

In the wire grip test, there was no significant difference between the HS and treatment groups in time of falling when compared to the Saline group in three consecutive trials (F (6, 56) = 0.3, Fig. 5). The harmaline-treated rats spent significantly less time on the rod than the Saline group (F (6, 56) = 0.79, *p* < 0.01, Fig. 5). HR rats spent a longer time on the rod when compared to the HS group (*p* < 0.05). These results showed that rats treated with Res exhibited a significantly longer duration on the rod, while HV and HRV groups (*p* < 0.05) showed significantly lower times when compared to durations exhibited by the HR group (Fig. 5).

### Harmaline-induced impairment of performance in the passive avoidance test was partially reversed by Res and VitD3

**Figure 6:**
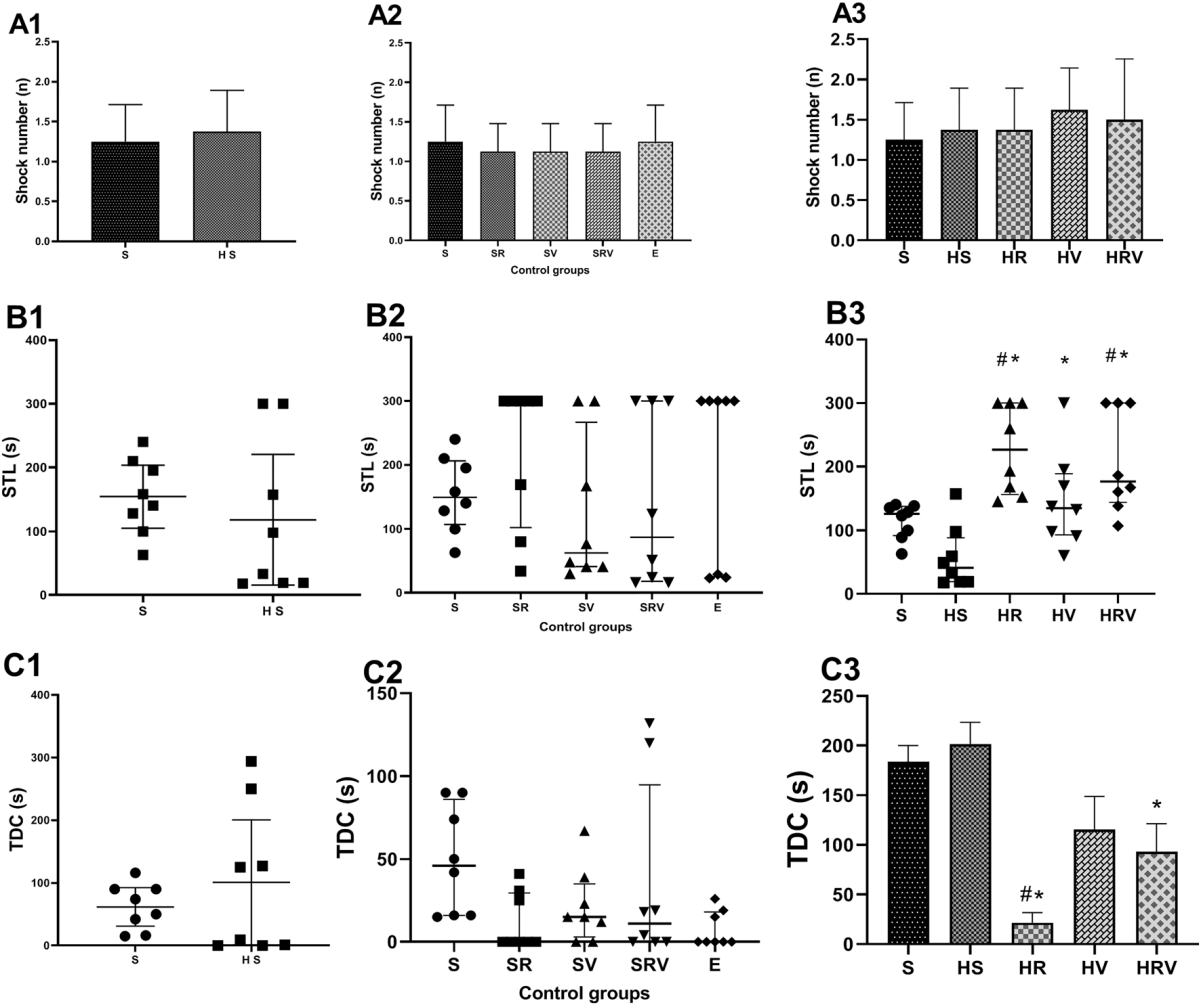
Passive avoidance memory analysis. (**A**) Shock number, (**B**) STL, and (**C**) TDC are presented for the different experimental groups. The HS group showed no significant difference in shock numbers compared to the Saline group. Data are expressed as the mean ± SD. The HR and HRV groups demonstrated an enhancement in STL compared to the Saline and HS groups. The HV group showed an increased STL compared to the group treated with harmaline. The HR and HVR groups spent less time in the dark chamber compared to the HS group. Data, which were not normally distributed are represented as medians with interquartile ranges as a scatter dot plot. (* in comparison to the harmaline group, # in comparison to the Saline group). Saline (S), Saline + Res (SR), Saline + VitD3 (SV), Saline + Res + VitD3 (SRV), Ethanol (E), harmaline + Saline (HS), harmaline + Res (HR), harmaline + VitD3 (HV), and harmaline + Res + VitD3 (HRV).

Statistical analysis revealed no significant difference in shock numbers in rats in the HS group as compared to numbers in the Saline group (F (4, 35) = 0.72, Fig. 6). Comparison of the STL showed that there was a significant enhancement in this parameter in the HR and HRV groups compared with the Saline and HS groups (F (4, 35) = 0.0001, *p* < 0.05). Additionally, the HS group treated with VitD3 showed an increased STL compared to the group treated with harmaline (*p* < 0.05, Fig. 6). The time spent in the dark chamber was also lower when the harmaline-injected rats were treated with Res and Res + VitD3 (F (4, 35) = 0.0001, *p* < 0.05) (Fig. 6).

### The effect of Res + VitD3 on Sirt1 and Lingo-1 mRNA expression

**Figure 7:**
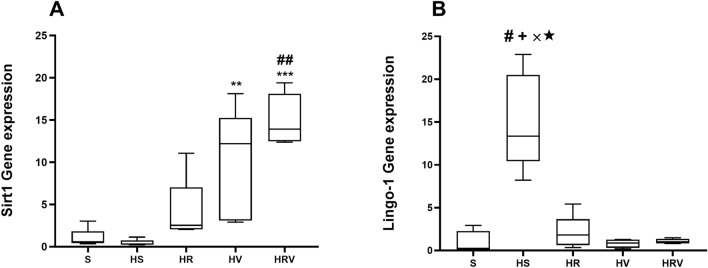
Real-time PCR expression data for (**A**) Sirt1 and (**B**) Lingo-1 are presented. No significant difference in Sirt1 expression was seen in the HS group compared to expression in the Saline and HR groups, but the expression was significantly higher in the HV and HRV groups. In addition, Lingo-1 expression was significantly higher in the HS group in comparison to levels in the HV, HR, HRV, and Saline groups. Data are represented using medians, with interquartile ranges shown as a box and maxima/minimum values represented as whiskers. (* in comparison to the harmaline group, # in comparison to the Saline group, × in comparison to the HRV group, + in comparison to the HV group, ★ in comparison to the HR group). Saline (S), harmaline + Saline (HS), harmaline + Res (HR), harmaline + VitD3 (HV), and harmaline + Res + VitD3 (HRV).

There was not a significant difference in Sirt1 expression in the HS group when compared to expression in the Saline and HR groups, but the expression was significantly higher in the HV and HRV groups (F (4, 20), *p* < 0.01, Fig. 7). In addition, Lingo-1 expression was significantly higher in the HS group when compared to levels in the HV, HR, HRV, and Saline groups (*p* < 0.001, Fig. 7).

### H&E staining

**Figure 8:**
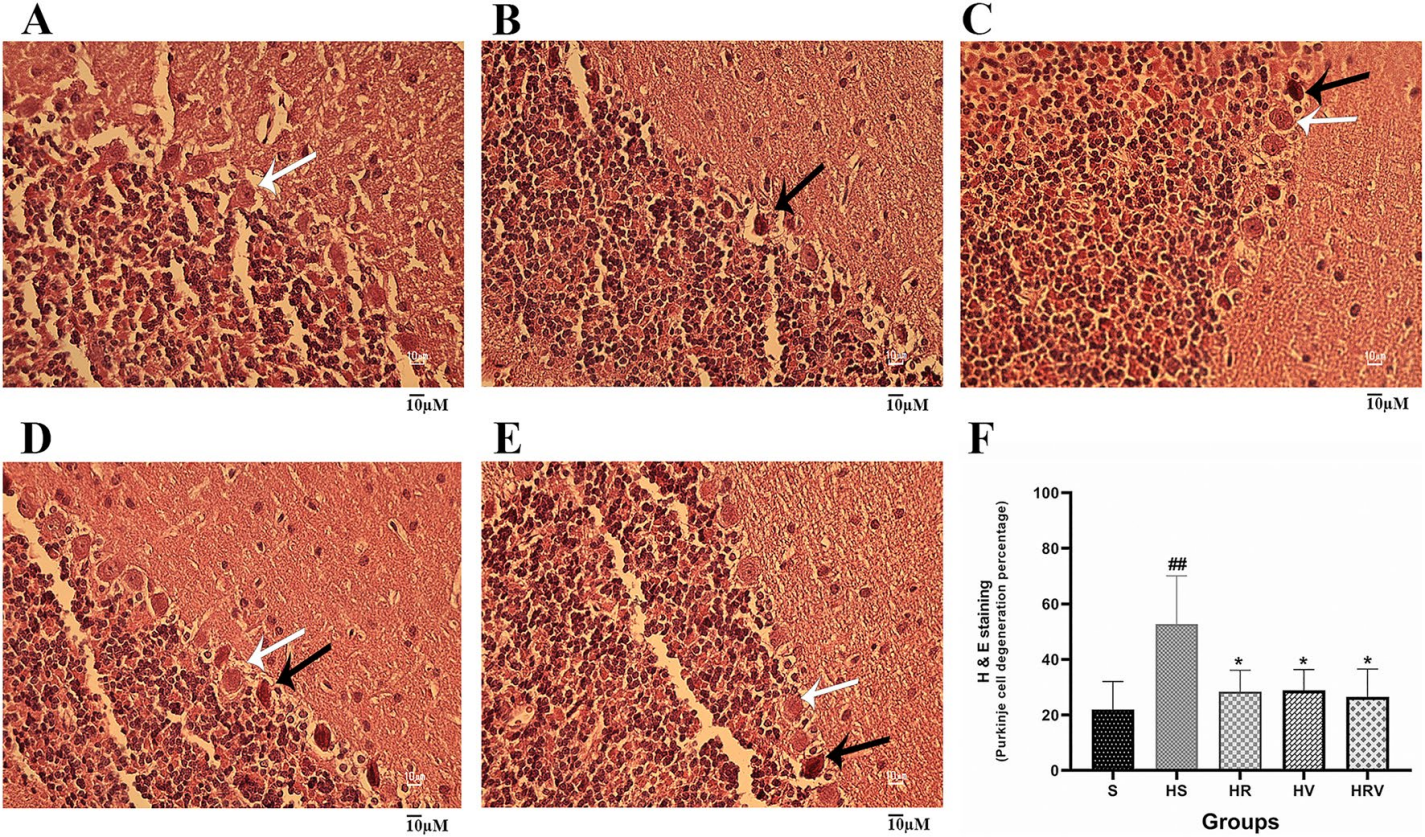
Histologic outcomes assessed by H&E staining. Results are shown across the (**A**) Saline, (**B**) HS, (**C**) HR, (**D**) HV, and (**E**) HRV experimental groups. (**F**) Quantification of Purkinje cells degeneration percentage in the cerebellum. White arrows indicate intact Purkinje cells and black arrows indicate degenerated Purkinje cells. Scale bar = 10 μm. Data are presented as mean ± SD. * in comparison to the harmaline group; # in comparison to the Saline group.

We preformed H&E histological tests to evaluate neuronal morphology features to determine effects of treatment on harmaline neurodegeneration. A notable neurodegeneration was observed in the harmaline group in comparison to the saline rats. Conversely, the HR, HV, and HRV groups exhibited a reduction in neurodegeneration, indicating a protective effect of VitD3 and Res on harmaline-exposed cells (Fig. 8).

### Nissl staining

**Figure 9:**
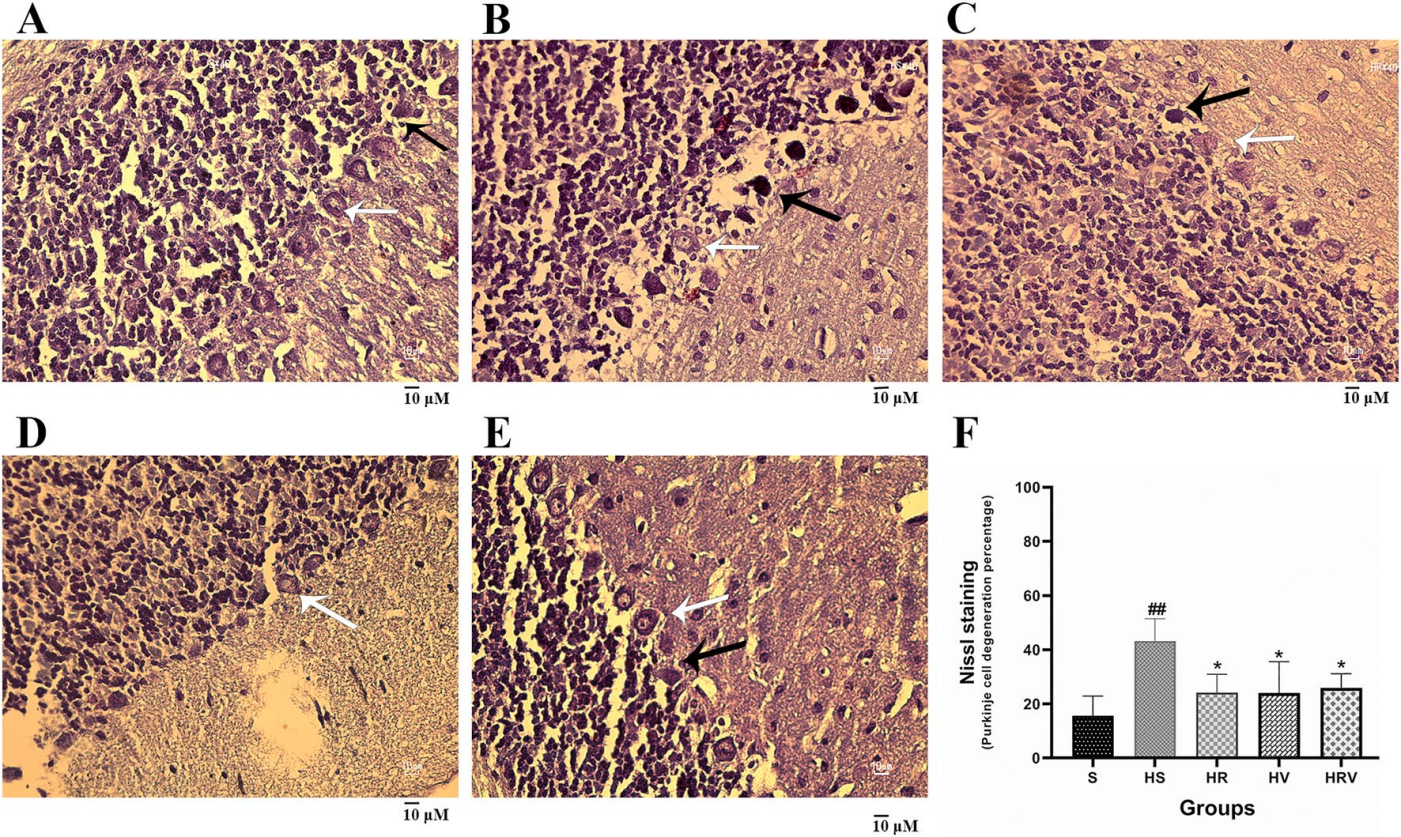
Nissl staining. Results from the (**A**) Saline, (**B**) HS, (**C**) HR, (**D**) HV, and (**E**) HRV experimental groups are presented. The HS group showed a significant increase in cell degeneration compared with the Saline group. The neurodegeneration percentage was significantly lower in the treatment groups compared to the HS group. Data are expressed as the mean ± SD (* in comparison to the harmaline group, # in comparison to the Saline group). White arrow shows intact Purkinje calls and black arrow shows degenerated Purkinje cells. (Magnification 400 ×). Saline (S), harmaline + Saline (HS), harmaline + Res (HR), harmaline + VitD3 (HV), and harmaline + Res + VitD3 (HRV).

As expected, analysis of the percentage of degeneration of cerebellar Purkinje cells showed that there was significantly higher cell degeneration in the HS group compared with Saline (*p* < 0.001). Indicating a neuroprotective effect, the neurodegeneration rate was significantly lower in the treatment groups (*p* < 0.05) compared to the HS group (Fig. 9).

### Ultrastructural findings

**Figure 10:**
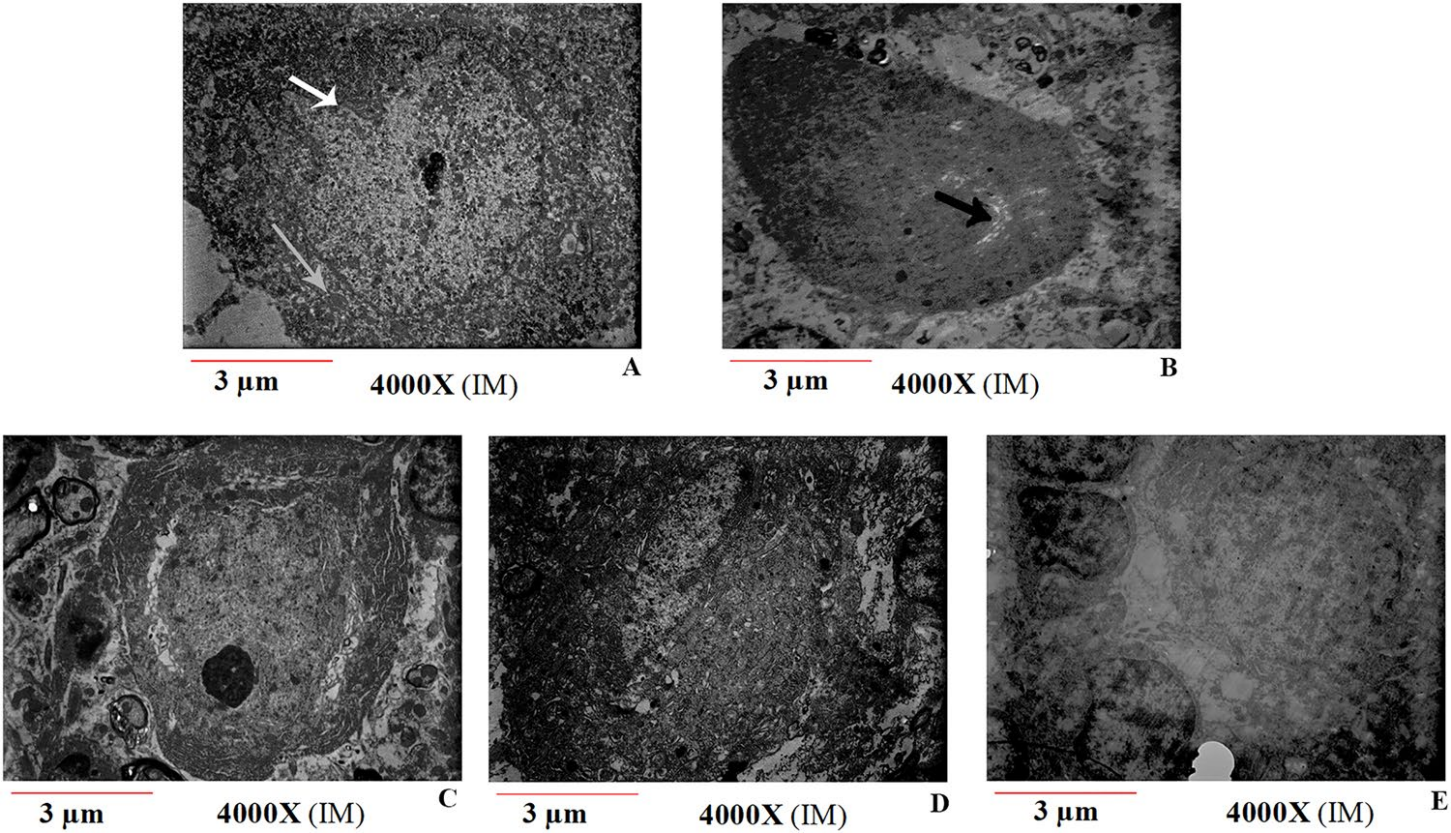
An electron micrograph of the ultrastructure of Purkinje cells in the cerebellum of rats. Results shown are from the (**A**) Saline, (**B**) HS, (**C**) HR, (**D**) HV, and (**E**) HRV experimental groups. Harmaline significantly altered the ultrastructure of Purkinje cells. The most common ultrastructural findings in harmaline group neurons were cell nuclear shrinkage, chromatin margination, and RER swelling. The ultrastructure of most Purkinje cells was preserved in the HR, HV, and HRV groups. The white arrows indicate intact cells with a distinct nucleus; the gray arrow indicates intact mitochondria; and the black arrows indicate RER swelling in degenerated cells. (4000x). Saline (S), harmaline + Saline (HS), harmaline + Res (HR), harmaline + VitD3 (HV), and harmaline + Res + VitD3 (HRV).

The ultrastructure of cerebellar Purkinje cells was investigated under TEM. In the Saline group, the neurons exhibited normal morphology, including a normal cell membrane, a distinct nucleus, dispersed chromatin, a prominent nucleolus, and intact organelles such as mitochondria and rough endoplasmic reticulum (RER) in the cytoplasm. Harmaline significantly altered the ultrastructure of Purkinje cells. The most common ultrastructural findings in harmaline group neurons were nuclear shrinkage, chromatin margination, and RER swelling. The ultrastructure of most Purkinje cells was preserved in the HR, HV, and HRV groups (Fig. 10).

## Discussion

Studies have provided encouraging data that VitD3 and Res could be beneficial for restoring normal movement and reducing postural and action tremor of the upper limbs, as well as tremor in the legs and trunk^48–53^. However, to the best of our knowledge, there have been no investigations of the effect of the combination of VitD3 and Res on ET. Therefore, in the present work, we examined the potential of VitD3 and Res drugs as therapeutics in a rodent neurotoxin-induced model of ET, the harmaline rat model by comparing and contrasting the effects of VitD3 and Res individually with actions when applied together. Exposure to harmaline results in impairment of the inferior olive nucleus and excitotoxicity of Purkinje cells, and induces acute postural and kinetic tremor of the limbs and trunk. Consistent with this, in our study, harmaline administration resulted in impaired motor coordination and was associated with a greater degree of Purkinje cell degeneration. The effects of VitD3 and Res on ET symptoms were examined, and behavioral tests evaluating motor control indicated that the co-treatment and separate application of VitD3 and Res reduced the severity of tremor in the ET model. However, our findings showed the application of the VitD3 and Res treatments did not have any effect on the abnormal gait and muscle strength impairment in ET rats, suggesting that using VitD3 and Res is not likely to improve all locomotor-based ET symptoms.

In addition to examining the effects of VitD3 and Res on motor-related behaviors in ET, we also explored cognitive behavioral alterations in the ET model. Our findings showed that, while we did not observe an effect on TDM in the open field arena, treatment with harmaline resulted in reductions in exploration, as evidenced by a decrease in rearing, which indicates an enhancement in anxiety-like behavior in this model. In our study, the decline in rearing seen in the ET model was improved by co-treatment with VitD3 and Res. We also examined effects on memory and found that the Res and co-treatment of VitD3 and Res ameliorated the impaired memory seen in the ET model.

Our results showing heightened anxiety-like behavior in our ET model as evidenced by reduced rearing are in line with those of Glynn et al. who showed reduced rearing in an ataxia model that exhibited tremor^54^. We found that while Res and VitD alone did not affect rearing, when combined, they had a positive effect on rearing, suggesting a reduction in anxiety-like behaviors. VitD3 has been linked to affective disorders in previous studies, and VitD3 deficiency is associated with an increased risk of developing depression and anxiety-like behavior in animals^55^. Additionally, it has been shown that VDR mutant mice displayed anxiety-like behaviors such as a decrease in vertical activity and an increase in grooming activity as measured in the open field test^55^. While the mechanism by which VitD3 could play a role in anxiety is unknown, neurons and glial cells that express VDR and VitD3-metabolizing enzymes are present in several different regions of the brain that control emotion, including the cortex, cerebellum, and limbic system. The the antioxidant, anti-inflammatory, pro-neurogenic, and neuromodulatory properties of VitD3 could lead to reductions in neuronal damage in these regions^56,57^. Furthermore, as affective disorders have been proposed to involve neuroinflammation, the positive modulation of antioxidant enzymes by calcitriol could play a role in reducing anxiety^58^. The ability of VitD3 to reduce psychopathology could involve the Sirt1 system^59^. VitD3 insufficiency was found to significantly reduce Sirt1 mRNA expression, while VitD3 supplementation restored Sirt1 transcription levels^60^. Another study reported that calcitriol attenuated neuroinflammation and induced neuroprotection in rodent models of Parkinson’s disease through upregulation of Sirt1^60^. Res is a natural polyphenol compound with multiple biological activities that has been reported to exert neuroprotective effects on several neurological disorders, including anxiety and depression^61^. The effects of Res on reducing anxiety-like behavior have been shown to involve the HPA axis^62^. Res has been detected to reduce pro-inflammatory factors such as cyclooxigenase 1 (COX1) which is involved in the production of cytokines^63^, nuclear factor kappaB (NF-kB)^64^, prostaglandins, NO, and TNF-α^65,66^. Additionally, Res has an effect on inflammation as well as sirtuin levels observed in anxiety. Res inhibits cytokine production by boosting Sirt1 concentrations, which have been shown to be associated with inhibiting negative psycho-behavioral outcomes^61,67–69^. In accordance with previous studies, our findings indicate that VitD3 and co-treatment with Res and VitD3 significantly raised Sirt1 levels. Taken together, the effects of VitD3 and Res on improving ET-associated anxiety-like behavior could involve the activation of the anti-inflammatory and Sirt1 pathways.

Our data indicated that although ET animals showed significantly impaired locomotor activity, there was no sign of improvement in rats treated with VitD3 and Res in terms of gait abnormality, muscle strength, or total distance moved. Our data are consistent with another study in which Res could not reverse locomotor impairments^70^. The effects of Res could be dose-dependent, and differences in this parameter could explain the discrepancy between our findings and those of earlier studies that showed an increase in locomotor activity. Therefore, the outcome of employing different Res doses should be considered.

Besides the effect of VitD3 on locomotor activity, several studies have proposed a regulatory role for VitD3 in cognitive memory and learning^71,72^, and VitD3 has been shown to have positive effects on cognitive symptoms in several neurodegenerative disorders such as Alzheimer’s disease^71^. By monitoring passive avoidance learning, we demonstrated that VitD3 improved ET-induced memory impairment. Becker et al. reported that prenatal VitD3-depleted rats exhibit alterations in learning and memory^72^. Furthermore, several studies indicated that VitD3 significantly ameliorated the cognitive dysfunctions present in fetal growth restriction rats and offspring born to mothers of advanced maternal age^73,74^. VitD3’s improvement of learning and memory could involve activation of Sirt1, and an increase in brain-derived neurotrophic factor (BDNF) levels^75^. Previous investigations have provided evidence that BDNF plays a pivotal role in neuronal survival. However, Lingo-1 can bind to the BDNF receptor, TrkB, and reduce its activation. Accordingly, limitations in the neuroprotective effect of BDNF could be due to the negative regulatory role of Lingo-1^76^. Tremors have been associated with the reduction of Ca^2+^ channels^77^, which could be due in part to Lingo-1 mediated deactivation of Ca^2+^ channels and a reduction in the membrane expression of these channels. Additional research should be conducted to evaluate the role of Lingo-1 mediated effects on TrkB receptors and Ca^2+^ channels in tremor. An additional potential mechanism could be that Lingo-1 activates the Nogo-66 receptor (NgR1) signaling pathway, which inhibits the development of oligodendrocytes and hinders myelination processes. Furthermore, Lingo-1 binds to the epidermal growth factor receptor (EGFR), which can lead to the downregulation of EGFR–PI3K–Akt signaling activity. Downregulation could be expected to reduce Purkinje cell survival and raise the risk of development of ET^78^.

The activation of Sirt1 by VitD3 has been observed to also strengthen the neuronal synapse, cause a decrease in AChE activity, reduce oxidative stress, and downregulate NF-kB, TNF-α, and IL-1^79–83^. Our results indicate that Res significantly improves memory deficits in ET rats, which is in line with previous studies^84,85^, including those that showed that Res improved performance in the passive avoidance task in a model of Alzheimer’s disease. Res was believed to exert its beneficial effects on impairments in recognition memory by enhancing activity in cholinergic systems, increasing levels of BDNF, facilitating phosphorylation of cAMP response element binding protein (CREB) signaling pathways in the prefrontal cortex, inhibiting the production of inflammatory cytokines, and reducing oxidative stress^84–89^. Recent studies have shown that Sirt1 plays a key role in cognitive performance and regulation of memory function through modulation of BDNF and inhibition of glutamate and the glutamatergic NMDA receptors, which are important factors in the differentiation of hippocampal neuronal cells and synaptic plasticity^90^. Moreover, Sirt1 stimulates the production of antioxidant enzymes and downregulates the expression of proinflammatory markers and apoptotic caspase 3 gene expression, which has been suggested to play a role in positive cognitive effects^91^.

The potent therapeutic effects of Res administration on motor impairments have been reported in animal models of Huntington’s disease, Parkinson’s disease, and in vitro amyotrophic lateral sclerosis (ALS) models^92^. Our study demonstrated that Res significantly increased the time of latency to fall and reduced the number of drops, resulting in amelioration of motor incoordination in the rotarod test. In line with our findings, several studies have indicated that Res can significantly improvement motor coordination during the rotarod test^93,94^. One plausible mechanism is the Nrf2 pathway, which facilitates cell survival in the cerebellum^92^.

In addition, Sirt1 has been shown to promote the neuroprotection of motor neurons through the modulation of several cellular pathways, including autophagy and mitochondrial biogenesis^95^. These studies confirmed our finding that Res-treated animals showed a marked increase in Sirt1 expression, leading to improved balance and coordination.

Sirt1 overexpression and activation play important roles in promoting motor nerve regeneration^96^. Furthermore, Res treatment improves motor behavior in motor-deficient mice through the activation of Sirt1^97^. Interestingly, Sirt1 neuroprotection involves the promotion of calcium homeostasis through transactivation of calcium regulatory genes. This mechanism could be particularly relevant in the cerebellum, as Purkinje cells are among the most metabolically active neurons and selectively express high levels of calcium-binding proteins^98^. Sirt1 regulates mitochondrial activity and reduces oxidative stress, mainly through the maintenence of peroxisome proliferator-activated receptor-γ coactivator-1α (PGC-1α) in a deacetylated state and promotion of a steady level of PGC-1α. This represents another potential mechanism by which Sirt1 could alleviate ET symptoms. Furthermore, other studies have indicated that Sirt1 might have a role in the regulation of neuroinflammation as it functions as a deacetylase for nuclear factor-kappa B, leading to a reduction in its transcriptional activity, suppression of inducible nitric oxide synthase expression, and decreased levels of tumor necrosis factor-alpha and interleukin-6^99^. Taken together, considering that Res improved motor activity and stimulated the Sirt1 pathway, the activation of Sirt1 may be a possible mechanism by which they improve motor activity. Therefore, therapies based on Sirt1 activation may offer a promising therapeutic approach for ET.

Although it has been shown that ET is also associated with enhancement of Lingo-1, to the best of our knowledge, this is the first study to report significant accumulation of Lingo-1 in the cerebellum of an animal model of ET caused by harmaline, which further validates the model for studying the mechanisms underlying ET. According to our results, Res, VitD3, and co-treatment of Res and VitD3 significantly decreased the transcription levels of Lingo-1, suggesting that reducing Lingo-1 levels as well as increasing Sirt1 activity results in improving ET symptoms.

As well as behavioral and molecular assessments, the current study examined the effects of Res, VitD3, and co-administration of Res,VitD3 on morphological and ultrastructural alterations caused by harmaline in a rat model of ET. Ultrastructural changes included cell nuclear shrinkage, chromatin margination, RER swelling, and heightened cell degeneration. Additionally, necrosis was evident in H&E and Nissl histological tests, which also revealed organelle swelling, plasma membrane rupture, cell lysis, and loss of structural integrity^100–104^. The excitotoxic effects of harmaline arise from activation and proliferation of Purkinje cells and their release of toxic substances such as interleukin-1b and nitric oxide, which in turn cause neuronal cell degeneration. Our histology findings showed that Purkinje cells were positively affected by Res, VitD3 and co-administration of Res and VitD3 as we found evidence for heightened protection from degeneration.

Co-administration of VitD3 and Res demonstrated notable effects on anxiety-like behavior and memory impairment in the ET model. VitD3 deficiency has been associated with increased risk for anxiety and depression in humans. The antioxidant, anti-inflammatory, pro-neurogenic, and neuromodulatory properties of VitD3 could contribute to reductions in anxiety by modulating various pathways, including Sirt1 and neuroinflammation. Similarly, Res has been reported to exert neuroprotective effects and reduce anxiety-like behavior through mechanisms involving the HPA axis, inhibition of pro-inflammatory factors, and modulation of Sirt1. The effects of VitD3 and Res may involve the activation of anti-inflammatory and Sirt1 pathways, leading to improvements in anxiety, memory, and motor coordination deficits observed in the ET model. Additionally, the co-administration of VitD3 and Res was associated with decreased transcription levels of Lingo-1, a protein implicated in ET pathology, suggesting a potential mechanism mediating reduction in ET symptoms. Although Res and VitD3 might be able to lessen ET symptoms on their own, the results of their combination treatment indicate that further research should be conducted to investigate the effectiveness of their co-administration for treatment of ET and associated neurological disorders.

## Conclusion

Our findings suggest that individually VitD3 and Res are effective in reducing tremor and anxiety-like behavior in the ET model; however, the effects of VitD3 and Res on motor and cognitive behaviors and protein levels was differential. Co-treatment with VitD3 and Res improved balance and coordination, and positive effects were associated with reductions in cell degeneration as well as increases in Sirt1 and decreases in Lingo-1. In our study, the combination of VitD3 and Res was shown to be a promising neuroprotective compound, likely due to antioxidant properties and the ability of these two factors to increase Sirt1 activity and decrease Lingo-1 levels. Consequently, it could be important to concurrently utilize both these treatments to leverage their complementary benefits and create a more comprehensive approach to alleviate the symptoms of ET. It is important to note that this does not necessarily imply a synergistic interaction between the two components; rather, it signifies that the combination results in effects on motor and cognitive behaviors, which would be expected to be more therapeutically adventageous than if each were used individually.

Although, promising results were obtained from the study examining the therapeutic potential of Res and VitD3 in an animal model of ET, it is essential to acknowledge the limitations associated with the application of these discoveries to human patients. The complex pathophysiology of human ET may not be entirely replicated in animal models, and variations in human anatomy, physiology, metabolism, and genetics may affect translation. Clinical research, particularly randomized controlled trials, is crucial to confirming the safety and effectiveness of VitD3 and Res in human ET populations. Such studies should address issues related to dose, length of treatment, patient selection, combination therapy, and tolerability. Moreover, another limitation of our study is the absence of an assay to quantify the amounts of Sirt1 protein. Given Sirt1’s potential role in ET, a test that measures Sirt1's protein level will be helpful for future studies to comprehend the possible interplay between Sirt1 and ET.

## Data Availability

The datasets used or analyzed during the current study are fully available and can be accessed by contacting the Corresponding author (shabani@kmu.ac.ir). A data repository URL (https://osf.io/qza4v/files/osfstorage) is also accessible.

## Abbreviations

ET: Essential tremor
Lingo-1: Leucine rich repeat and Ig domain containing Nogo receptor interacting protein-1
VitD3: 1,25-Dihydroxyvitamin D = vitamin D3 = Calcitriol
Res: Resveratrol
Sirt1: Silent Mating Type Information Regulation 2 Homolog1 = Sirtuin 1

## Code availability

All software applications used are included in this article.
